# Biomolecular condensates form spatially inhomogeneous network fluids

**DOI:** 10.21203/rs.3.rs-3419423/v1

**Published:** 2023-10-18

**Authors:** Furqan Dar, Samuel R. Cohen, Diana M. Mitrea, Aaron H. Phillips, Gergely Nagy, Wellington C. Leite, Christopher B. Stanley, Jeong-Mo Choi, Richard W. Kriwacki, Rohit V. Pappu

**Affiliations:** 1 Department of Biomedical Engineering and Center for Biomolecular Condensates, Washington University in St. Louis, St. Louis, MO 63130, USA; 2 Center of Regenerative Medicine, Washington University in St. Louis, St. Louis, MO 63130, USA; 3 Dewpoint Therapeutics Inc., 451 D Street, Boston, MA 02210, USA; 4 Department of Structural Biology, St. Jude Children’s Research Hospital, Memphis, TN 38105, USA; 5 Neutron Scattering Division, Oak Ridge National Laboratory, Oak Ridge, Tennessee 37831, USA; 6 Computational Sciences and Engineering Division, Oak Ridge National Laboratory, Oak Ridge, TN 37830; 7 Department of Chemistry and Chemistry Institute for Functional Materials, Pusan National University, Busan 46241, Republic of Korea; 8 These authors contributed equally: Furqan Dar, Samuel R. Cohen, and Jeong-Mo Choi

## Abstract

The functions of biomolecular condensates are thought to be influenced by their material properties, and these are in turn determined by the multiscale structural features within condensates. However, structural characterizations of condensates are challenging, and hence rarely reported. Here, we deploy a combination of small angle neutron scattering, fluorescence recovery after photobleaching, and bespoke coarse-grained molecular dynamics simulations to provide structural descriptions of model condensates that mimic nucleolar granular components (GCs). We show that facsimiles of GCs are network fluids featuring spatial inhomogeneities across hierarchies of length scales that reflect the contributions of distinct protein and peptide domains. The network-like inhomogeneous organization is characterized by a coexistence of liquid- and gas-like macromolecular densities that engenders bimodality of internal molecular dynamics. These insights, extracted from a combination of approaches, suggest that condensates formed by multivalent proteins share features with network fluids formed by associative systems such as patchy or hairy colloids.

Biomolecular condensates are compositionally-defined membraneless bodies that enable spatiotemporal organization and control over a range of biochemical reactions in cells ^1, 2, 3, 4, 5, 6^. Known condensates in cells comprise specific combinations of nucleic acids and proteins ^7, 8^. Condensates are often thought of as spatially homogeneous liquids that form via liquid-liquid phase separation (LLPS) ^9, 10^. However, a more nuanced view has begun to emerge, and this is driven by realization of the importance of multivalence as a driver of condensate formation ^11^. As a result, there is growing consensus that condensates are viscoelastic materials ^12, 13, 14, 15, 16, 17, 18, 19, 20, 21^ that form via coupled associative and segregative phase transitions of associative macromolecules ^22, 23^.

Proteins that are exemplars of associative macromolecules have distinctive molecular grammars, typically featuring oligomerization domains, multiple ligand binding domains, and intrinsically disordered regions (IDRs) with distinctive sequence features ^22, 24, 25^. The coupling of associative and segregative transitions, referred to as COAST ^22^, and the driving forces for these transitions derive from the molecular grammars of associative macromolecules ^20, 26, 27, 28, 29, 30, 31, 32, 33, 34, 35, 36^. Complex coacervation is the clearest illustration of the coupling between associative and segregative phase transitions ^37, 38, 39, 40, 41, 42^. Other instantiations of COAST-like processes include the coupling of percolation and phase separation ^11, 27, 43, 44^. Percolation, specifically bond percolation, also known as physical gelation ^35, 44, 45, 46^, is a continuous associative transition whereby motifs or domains known as stickers form reversible, physical crosslinks to enable the formation of sequence- and architecture-specific networks that span the length scale of the system of interest ^32, 43^. As the networks grow, phase separation can be driven by the balance of inter-macromolecule, macromolecule-solvent, and solvent-solvent interactions, controlled largely by the sequence and structural characteristics of spacers, which are regions outside stickers ^47, 48^.

COAST-like processes give rise to condensates with network-like internal organization ^28, 49, 50, 51^. The topological characteristics of condensate-spanning networks will be defined by the architectures of the constituent molecules and the extent of crosslinking among the molecules ^32, 43, 49, 52^. The internal viscosity of condensates and the elasticity of the networks will be governed by the interplay between the timescales for molecular transport within and into / out of condensates and the timescales for making and breaking physical crosslinks. Furthermore, network-like internal organization will engender spatial inhomogeneities of macromolecular crosslinks and densities ^22, 26, 28, 50^.

Viscoelastic materials have time-dependent properties and network structures contribute directly to viscoelastic moduli of condensates ^53^. Even if condensates are terminally viscous fluids, there will be short timescales where the materials are dominantly elastic ^50^. Condensates can age, and if they undergo equilibrium fluid-to-solid transitions, then the terminal states are those of elastic solids ^50^. Alternatively, some aged condensates can behave like viscoelastic network glasses ^54^. While the network-like organization within condensates has been inferred from viscoelastic measurements and validated by the reproduction of measured moduli using computed network structures ^50^, there is a paucity of direct measurements of network structures within condensates.

At least two types of approaches have been used to probe the internal organization of condensates. A recent study used single fluorogen imaging that leverages the use of freely diffusing, environmentally sensitive, solvatochromic dyes ^55^. The spatial localization and orientational preferences of these dyes can be gleaned using single molecule approaches and used to reconstruct the internal organization of condensates. An alternative approach, rooted in its historical use in the study of simple and complex fluids, is based on scattering measurements, specifically small angle neutron scattering (SANS) ^56, 57, 58, 59^. A key advantage of SANS is that one can probe for the presence of spatial inhomogeneities that range from a few angstroms to hundreds of nanometers ^60^.

Here, we direct our studies of liquid-state structure to condensates that are mimics of nucleolar sub-phases. The nucleolus is a spatially organized condensate featuring at least three coexisting sub-phases. The GC, which is the outermost layer, is scaffolded by nucleophosmin (NPM1) ^61, 62^. Condensates formed by the complexation of NPM1 and Arginine-rich (R-rich) peptides and proteins such as rpL5 and SURF6 have been used to advance the molecular handoff model for ribosomal subunit assembly, which occurs within the nucleolar GC ^7, 8, 63, 64, 65, 66^. Measurements of internal structure within condensates that are based on SANS were first reported by Mitrea et al., ^63^. They studied condensates formed via heterotypic interactions of cationic, arginine-rich peptides (rpL5) and N130, the N-terminal 130-residues of NPM1, which includes its oligomerization domain (OD) and at least three distinct short regions that are rich acidic residues ^67^. These condensates serve as facsimiles of nucleolar GCs ^8, 63, 64^.

How might liquid-state theory be adapted for describing condensates? Pioneering advances in describing the structures of simple Van der Waals liquids and complex, associative liquids formed by small molecules, colloids, and macromolecules have been made over several decades by integrating scattering or diffraction data with computer simulations ^68, 69, 70, 71, 72, 73, 74, 75^. Here, we follow this lead by adapting well-established approaches and integrating them with graph theoretic methods to arrive at experimentally anchored, and computationally driven descriptions of network fluid structures of condensates formed by N130 and rpL5. The internal structure and dynamics of condensates formed by interactions among N130, and Arg-rich peptides were probed using SANS and fluorescence recovery after photobleaching (FRAP). The data were interpreted using simulations based on bespoke, sequence-specific coarse-grained models. The latter were developed using a machine-learning approach that bootstraps against atomistic simulations of small numbers of molecules ^76^.

The approaches developed and deployed here show that condensates formed by N130 and rpL5 (N130+rpL5) have short-range order, long-range disorder, and a layering at intermediate length scales with all the hallmarks of fluid-like organization. From quantitative analysis of structure factors and computed radial distribution functions we estimate the mean coordination number in the first shell to be akin to that of water, an archetypal small molecule network fluid. Graph-theoretic analysis shows that there are two sub-graphs that describe the totality of structure of the network fluid. One of the sub-graphs is gas-like, and the other is liquid-like. This gives rise to two distinct classes of motions, as probed in terms of the mean squared displacements (MSDs) of the OD of N130: one of the dynamical modes is super-diffusive (gas like) and the other is sub-diffusive (akin to what we would expect in a viscoelastic, terminally viscous fluid). The overall picture that emerges from the blend of experimental and computational analyses is of a network fluid with structural and dynamical features that are akin to fluids formed by associative molecules such as patchy / hairy colloids.

## RESULTS

### N130 and rpL5 form condensates via complexation

**Fig. 1:**
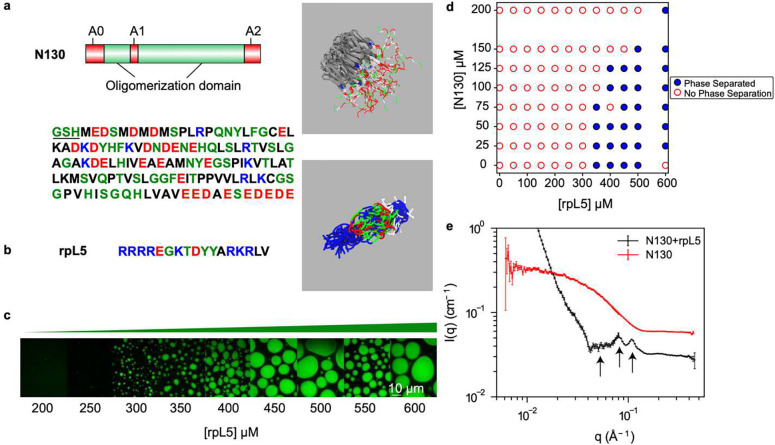
Complexation between acidic regions within N130 and R-motifs of rpL5 is required for condensation. **(a)** Schematic representation of N130 including the different acidic regions. The amino acid sequence of N130 is also shown. The three acidic regions A0 (see text), A1, and A2 span residues 4–18, 35–44, and 120–133, respectively. The underlined N-terminal residues GSH are cloning artefacts. On the right, we show the overall structure of the hairy colloid generated by superposition of 50 distinct conformations from all-atom simulations. The OD (PDB ID 4N8M), in gray, was modeled as a rigid molecule in the atomistic simulations. **(b)** The sequence of rpL5. The panel on the right shows a superposition of 50 different conformations extracted from atomistic simulations based on the ABSINTH model. **(c)** Confocal microscopy images of phase separation of 100 μM N130 upon titrating the concentration of rpL5 in buffer and 150 mM NaCl. N130 is labeled with AlexaFluor488. **(d)** Two-component phase boundary for N130+rpL5, showing the result of concentration titrations. **(e)** SANS curve showing the intensity *I*(*q*) plotted against *q*, the scattering vector, for condensates formed by a N130 (200 μM): rpL5 solution at 1:3 stoichiometry. A fitting analysis, described in the Methods, gives peaks at scattering vectors corresponding to 55 Å (left arrow), 77 Å (middle arrow), and 119 Å (right arrow). The SANS curve for N130 alone is shown for comparison. In the interest of clarity, this curve is shifted upwards by 0.03 cm^−1^.

The N130 construct corresponds to residues 1–130 of mouse NPM1 that includes the OD interspersed by short, disordered regions (Fig. 1a). Previous work showed that N130 forms condensates with R-rich peptides which serve as minimal facsimiles of the nucleolar GC ^63^. Two regions within N130 are enriched in acidic residues. One encompasses a flexible loop (residues 35–44, termed A1). The other is located at the C-terminus (residues 120–130, termed A2). These regions were shown to mediate interactions with R-rich peptides and promote condensate formation (Fig. 1a) ^67^. There also is an N-terminal region with acidic character (residues 1–16, termed A0).

We first performed atomistic simulations using the ABSINTH implicit solvation model and forcefield paradigm ^77^. In these simulations, the OD was modeled as a rigid domain, and conformations adopted by the IDRs were sampled using Monte Carlo moves. From the simulations, we obtained an overall structure of the N130 pentamer that is reminiscent of hairy colloids ^78^, featuring disordered, acidic regions that protrude from one side of the pentamer. This topology is a specific instantiation of patchy / hairy colloids that are known to form network fluids through anisotropic interactions engendered by the architectures of the constituent molecules ^79, 80, 81, 82, 83^. N130 forms condensates with R-motif-containing disordered proteins including peptides such as rpL5 (Fig. 1b) ^63^. The rpL5 peptide was taken from the ribosomal protein L5 and its sequence corresponds to the region that has been shown via experiments to interact with NPM1 ^67^. Simulations show that rpL5 adopts ensembles of expanded conformations that maximize the favorable solvation of Arg and Lys residues ^84^.

Titrating in rpL5 at a fixed N130 concentration of 100 μM gives a threshold rpL5 concentration for phase separation that is between 250 and 300 μM rpL5 (Fig. 1c). The phase boundary (Fig. 1d), mapped by delineating the boundary between the one- and two-phase regimes by titrating the concentrations of N130 and rpL5, is consistent with previous experimental studies ^63, 85.^

Next, we performed SANS measurements to probe the molecular organization within condensates formed by N130 complexed with rpL5 (Fig. 1e). The SANS intensity is a convolution of the form factor and structure factor. The former quantifies the scattering that results from the average shapes of the scatterers, whereas the latter quantifies how the particles scatter neutrons due to the spatial correlations caused by intra- and intermolecular interactions. Specifically, the structure factor measures density correlations in reciprocal space, whereas the form factor is the Fourier transform of the density distribution.^86^.

The importance of complexation as a driver of internal organization is made clear by the lack of peaks in the structure factor for N130 when rpL5 is absent from the solution. The inhomogeneities in spatial densities that are evident in the scattering profile (shown by the arrows of Fig. 1d) are indicative of order on different length scales. Fitting analysis, described in the Methods, shows peaks at scattering vectors corresponding to length scales of 55 Å, 77 Å, and 119 Å (Fig. 1e). Here, the molecular diameter of the OD (~53 Å) is a useful ruler for calibrating different length scales. The averaging process inherent in the experimental data obscures details of the ordering. Accordingly, to further characterize the nature of the ordering and the interactions that contribute to ordering, we turned to computational approaches. This approach is reminiscent of classical methods developed to study liquid state structure.

### Systematic coarse-graining and predictions

To investigate the internal structure of fluid-like condensates, we performed coarse-grained (CG) simulations of the N130+rpL5 condensates. In the CG model, the OD of the N130 pentamer was modeled as a single, spherical bead defined by excluded-volume interactions. We used a single-bead-per-residue representation for the residues in the IDRs of N130. Accordingly, in addition to the acidic regions, A1 and A2, we also explicitly modeled the N-terminal region of N130 (termed A0). All residues in rpL5 were modeled as single beads.

**Fig. 2:**
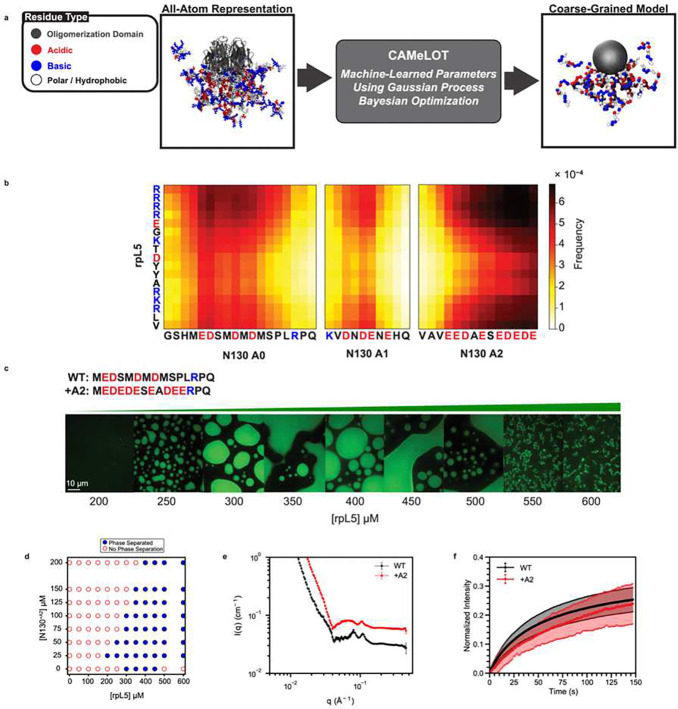
Coarse-grained simulations of N130+rpL5 condensates highlight the importance of an N-terminal acidic region (A0) within N130. **(a)** Coarse-graining procedure for N130 and rpL5 systems. To generate sequence- and system-specific CG models for N130 and rpL5 peptides, we start with atomistic simulations of the individual molecules, using CAMPARI (http://campari.sourceforge.net) and ABSINTH ^77^. We then prescribe a CG model for the system. Here, the residues of the OD are collectively modeled as one large bead depicted here in gray. Next, for regions outside the OD, the residues are single beads, and the bead types are organized into three groups: 1: E,K,R,D; 2: V,F,M,I,L,Y,Q,N,W,H; and 3: A,P,G,S,T,C, respectively. To determine the optimal interaction parameters for the CG model, we use a machine-learning-based approach using CAMELOT^76^. **(b)** Bead-to-bead contact maps from coarse-grained simulations of dense-phases comprising a 1:3 ratio of N130 and rpL5. The A1 and A2 regions interact favorably with rpL5 peptides. The contact maps reveal new, hitherto unappreciated interactions involving a region we refer to as A0. **(c)** Confocal microscopy images of phase separation at a fixed concentration of N130^+A2^ upon titrating with rpL5. The A0 tract in the wild type is replaced with the reversed sequence of the A2 tract. (**d**) Two-component phase boundary for N130^+A2^+rpL5, showing the result of concentration titrations. (**e**) SANS curves show that a broad peak forms at lower *q* ranges, with the shift implying a shift toward extended conformations in rpL5-induced droplets of the +A2 mutant compared to wild type. SANS data were collected with 200 μM N130 and 600 μM rpL5. For clarity, the curve for N130^+A2^+rpL5 is shifted upwards by 0.03 cm^-1^. **(f)** FRAP curves for the condensates containing N130 and N130^+A2^, along with the measured recovery times. FRAP curves were collected at 100 μM N130 and 400 μM rpL5 with twelve replicates. Error bars represent the standard deviation of the mean.

The systematic coarse-graining procedure was initiated by bootstrapping against information generated using atomistic simulations based on the ABSINTH implicit solvation model and forcefield paradigm ^77^ (Fig. 2a). Having prescribed the resolution for the CG model, we then use the ensembles from atomistic simulations to generate forcefield parameters for the CG model. For this, we use the CAMELOT algorithm ^76^ that combines a Gaussian Process Bayesian Optimization ^87^ module, with an appropriate architecture and CG model. The optimal parameters derived from CAMELOT serve to minimize the difference between the atomistic conformational ensembles and the CG representation. The CG model enables the use of less computationally intensive models while also capturing system- and sequence-specific effects. Further, the CG representation allows us to simulate the dense phase using approximately 100 copies of N130 and 1500 copies of the rpL5 peptide, thereby minimizing effects due to finite size, which can be significant when fewer than ten macromolecules are used to model dense phases ^42^. The stoichiometric ratios and the macromolecular concentrations used here correspond to the system being in the two-phase regime, such that the entity being simulated is a computational facsimile of the dense phase formed by N130+rpL5.

Results from the coarse-grained simulations of dense phases were used to compute inter-residue contact maps between the disordered regions of N130 and the rpL5 peptide (Fig. 2b). As expected, the A1 and A2 regions make several favorable contacts with the basic residues in rpL5. We also observed that the A0 region makes contacts with the basic residues in rpL5. The frequency of contacts suggests that this IDR forms stronger interactions with rpL5 than A1. The interactions appear to be primarily electrostatic in nature, involving four acidic residues within A0. The contact maps derived from the coarse-grained simulations suggest a rank ordering of interactions between the acidic regions and rpL5, with A2 being the most favorable and A1 the least.

Our predictions motivated the generation of a new mutant construct where we replaced A0 with the residues from, in reverse order, to increase the linear charge density. We refer to this construct as the N130^+A2^ mutant. This construct has more acidic residues than wild type. We proposed, based on the simulations, that the +A2 mutant should form condensates with a lower threshold concentration of rpL5 for a given N130 concentration. Indeed, titrating in rpL5 at a fixed concentration of N130^+A2^ leads to a lower rpL5 threshold concentration (Fig. 2c) when compared to the threshold concentration that is required for condensation with wild-type N130 (Fig. 1c). Increasing the strength of the electrostatic interactions in A0 reduces the threshold concentration for rpL5 from 300 μM to below 250 μM at 100 μM N130. Note that the designs were chosen to ensure that the stoichiometric ratio required for condensation does not change. Similar phase behaviors were previously reported for complex coacervating systems ^85, 88, 89^. The phase boundary (Fig. 2d), mapped by titrating the concentrations of N130^+A2^ and rpL5, is consistent with this observation.

We investigated the impact of the +A2 mutant using SANS (Fig. 2d). We observe a pronounced peak at intermediate *q*-values and a slight shift of the peak at ~10 Å^−1^ to lower *q*-when compared to N130. This change can be explained in terms of increased electrostatic repulsions in the disordered N- and C-termini of N130 emanating from the same face of the OD ^67^. The C-terminus of the wild-type protein contains nine negatively charged residues corresponding to A2, and the +A2 mutant increases the net charge on the pentamer by 25.

We also measured the impact of the +A2 mutant on the dynamics of N130+rpL5. For this, we performed measurements of fluorescence recovery after photobleaching (FRAP) on the condensates (Fig. 2e). The FRAP curve for N130+rpL5 indicates dynamical exchange with the bulk solution with the recovery time constant being 53 ± 2 s. Increasing the total charge on N130 via the +A2 mutant decreases the overall extent of FRAP, resulting in a significantly longer recovery time of 103 ± 8 s. Similarly, we observe that N130^+A2^ + rpL5 displays slower overall dynamics at shorter timescales, and the dynamics of the two systems approach one another at longer times. The slower dynamics observed for the system with N130^+A2^ are expected for a system with stronger driving forces for condensation and stronger molecular interactions. The totality of the data, driven by predictions from simulations, show that the N-terminal disordered region of N130 houses an A0 acidic tract that contributes to the phase behavior of N130+rpL5 mixtures.

### Condensates formed by N130+rpL5 are network fluids

As observed in the SANS data (Fig. 1e), N130+rpL5 condensates display correlations at length scales that are consistent with dimensions of the OD of N130. Therefore, we focus our analysis of the liquid-state structure on the spatial correlations formed by N130. Obtaining the experimental structure factor by deconvolution of the SANS spectrum would require modeling the form factor. This is a complicated task that becomes intractable given the geometry of the molecules, which are far from simple spheres ^90^. Instead of treating the determination of the structure of N130+rpL5 condensates as an inverse problem, we derived liquid-state structure from simulations by computing pairwise correlations in real space via the radial distribution function g(r). This is the real-space analog of the experimentally measurable structure factor ^91^. It describes how spatial densities change as a function of distance from an arbitrary reference particle. Normalized to an ideal gas, where the distance between particle pairs is completely uncorrelated, g(r) is the standard descriptor of liquid structure in theory, experiment, and simulations.

**Fig. 3:**
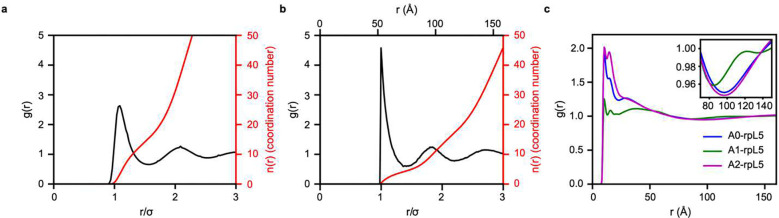
Radial distribution functions affirm the network fluid structure of N130+rpL5 condensates. **(a)** Radial distribution function showing the correlations between particles in a Lennard-Jones fluid along with the volume integral *n*(*r*) quantifying the mean coordination number as a function of interparticle separation *r*. On average, approximately twelve Lennard-Jones particles are found within the first coordination shell. **(b) g(r)** in black and *n*(*r*) in red quantifying the correlations between the ODs of N130 in the simulated N130+rpL5 system. There are local maxima at 53 Å, 95 Å, and 140 Å. These are in accord with the peaks in SANS profiles. On average, approximately four oligomerization domains are found within the first coordination shell. This number is consistent with a network fluid, such as water. **(c)** g(r) quantifying the correlations between negative charges in the acidic regions of N130 and the positive charges in rpL5. W(r)=−RTlng(r) quantifies the free energy change associated with bringing a pair of sites to a distance r from one another. From the site-site g(r), it follows that the free energy change is between −0.5 and 0 kcal/mol over a distance range of 50 Å. These interactions are strongest between A2 and rpL5 and weakest between the A1 tract and rpL5. Averages were calculated over 3 replicates for the Lennard-Jones systems and over 5 replicates for the N130.

To calibrate our expectations, we first computed g(r) (Fig. 3a) for the Lennard-Jones fluid. This, as has been well-established, has all the characteristic features of a simple liquid defined purely by packing considerations ^92^. The first peak corresponds to the nearest neighbors in the vicinity of the reference particle of diameter σ, and the additional peaks correspond to higher-order neighboring shells. Because g(r) is a measure of the density correlations, it quantifies how the average density at a separation r from the center of any particle varies with respect to the average density of the fluid. The density correlations are large in the vicinity of the reference particle, and the probability of determining particle positions decays as a function of distance until the density becomes indistinguishable from the average density of the fluid ^93^.

The liquid state structure can be further characterized by the volume integral over g(r) up to defined positions such as the first minimum. This quantifies the nearest-neighbor coordination number ^91^. For the Lennard-Jones fluid, the coordination number is approximately twelve due to optimal packing of the spherical particles. In contrast, more complex liquids like water have open, network-like organization due to hydrogen bonds that result in less efficient packing. This results in the tetrahedral organization of liquid water, which has a nearest-neighbor coordination number of approximately 4.5 with respect to the oxygen-oxygen g(r) ^94^.

From the coarse-grained simulations, we compute the g(r) between pairs of ODs of different N130 molecules (Fig 3b). The g(r) computed between pairs of ODs of N130 yields a coordination number of approximately four. This suggests that N130 forms a network fluid that resembles the open structure of liquid water. The peaks at 52 Å, 100 Å, and 145 Å correspond to ordering beyond the molecular length scale. The positions of the peaks are in reasonable agreement with the molecular spacing indicated in our SANS data. The positions also further suggest that most of the intensity in the SANS measurements comes from the organization of the ODs inside the condensate.

Next, we computed the g(r) between basic residues in rpL5 and acidic residues in the three different regions of N130. Note that these g(r) profiles were computed as a linear superposition of pair distributions between all the basic residues in the peptide and all the acidic residues in a specific region. Each of these g(r) profiles has distinct peak positions and heights for the first maximum. The heights of the peaks, realized in a range of *r* < 50 Å, are highest for A2 and lowest for A1. The computed profiles suggest the existence of an interaction hierarchy that is distributed among the acidic regions. Indeed, these trends are observed in the corresponding potentials of mean force (Supplementary Fig. 1). The most favorable interactions in the distance range of *r* < 50 Å are realized between A2 and rpL5. The hierarchy of interactions encoded in the different acidic tracts of N130 agrees with the contact maps (Fig. 2b). These patterns are a direct consequence of the hairy colloidal architecture of the pentameric N130 protein.

### Interactions between acidic regions and rpL5

**Fig. 4:**
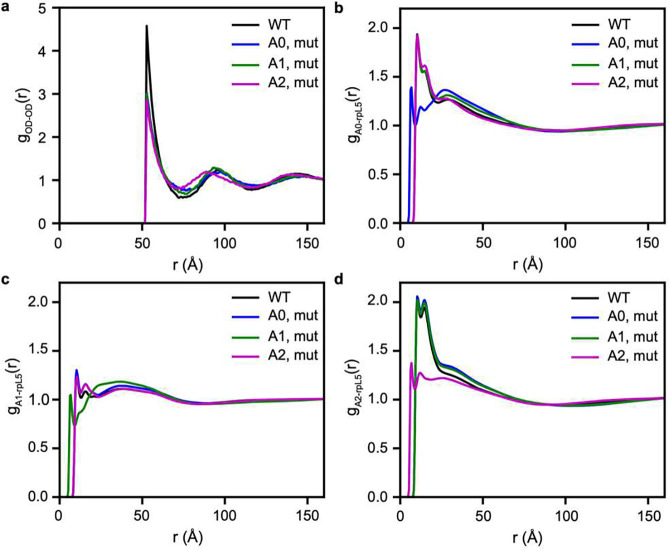
Interactions between acidic tracts and rpL5 are modular and independent of one another. **(a)** *g*_OD-OD_(*r*) computed after neutralizing the charges in each of the acidic tracts indicated in the legend. **(b)** *g*_A0-rpL5_(*r*) quantifies the pair correlations between acidic residues in A0 and basic residues in rpL5. Results are shown for the WT (black), when acidic residues are neutralized in A0 (blue), A1 (green), and A2 (magenta). Neutralizing charges within A0 weakens the interactions between A0 and rpL5. However, neutralizing the charges within A1 and A2 does not significantly affect the interactions between A0 and rpL5. **(c)** *g*_A0-rpL5_(*r*) quantifies the pair correlations between acidic residues in A1 and basic residues in rpL5. Results are shown for the WT (black), when acidic residues are neutralized in A0 (blue), A1 (green), and A2 (magenta). Neutralizing charges within A1 weakens the interactions between A1 and rpL5 (green curve). However, neutralizing the charges within A0 and A2 does not significantly affect the interactions between A1 and rpL5. **(d)** *g*_A2-rpL5_(*r*) quantifies the pair correlations between acidic residues in A2 and basic residues in rpL5. Results are shown for the WT (black), when acidic residues are neutralized in A0 (blue), A1 (green), and A2 (magenta). Neutralizing charges within A2 weakens the interactions between A2 and rpL5 (magenta curve). However, neutralizing the charges within A0 and A1 does not significantly affect the interactions between A2 and rpL5. In all cases, averages were calculated over 5 replicates.

Next, we computationally neutralized the charges in each of the acidic tracts while keeping the density of the simulated dense phases fixed to that of the wild type N130+rpL5 condensates. These simulations were designed to assess how mutations that affect the electrostatic interactions mediated by one region affect the totality of the network structure. We computed g(r) between pairs of ODs and between the acidic regions on N130 and basic residues of rpL5. Neutralizing the acidic residues on any of the three regions significantly alters the overall network structure. This is seen as a reduction in the first maximum of *g*_OD-OD_(*r*) (Fig. 4a). The magnitude of the reduction in the first maximum is greatest for the A2 mutant, followed by the A0 and A1 mutants, for which the values of the peak heights are statistically similar within error (Supplementary Fig. 2). This indicates that mutations to A2 affect the overall structure more than mutations to the other regions. The potentials of mean force (Supplementary Fig. 3) corroborate this inference showing that the least favorable free energy is between the oligomerization domains of the A2 mutant. The interactions between basic residues of rpL5 and the acidic residues within A0, A1, and A2 are modular. This is clear from the *g*_AX-rpL5_(*r*) profiles, where X = 0, 1, or 2, that we compute from simulations where one of A0, A1, or A2 is neutralized. The *g*_AX-rpL5_(*r*) profile deviates from that of the WT only for the region in which the charges are neutralized. Otherwise, the profiles remain roughly equivalent to those obtained from the wild-type N130+rpL5 condensates. This suggests that the acidic regions make modular, roughly independent interactions with freely diffusing rpL5 peptides.

The *in-silico* experiments enable us to unmask the contributions of different acidic regions within N130 to interactions with rpL5 within condensates. For these experiments, we fixed the density of the dense phase, and this allowed us to compare the interactions of different regions to one another. Performing such analyses *in vitro* is difficult, with there being numerous complications including challenges of protein preparation and the fact that changes to the acidic tracts, as designed in the *in-silico* investigations, would likely alter phase behavior. Further, while synthetic mutations can be made without changing any other interactions *in silico*, this is impossible to achieve *in vitro*.

### Effect of the Arg motifs of rpL5 on network fluid structure

**Fig. 5:**
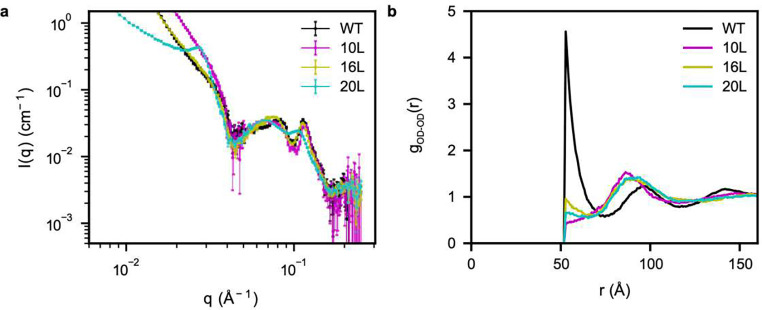
Linkers between R-motifs in rpL5 and changes to the charge of A2 influence the local structure of the network fluid. (a) SANS curves for N130 with each of the synthetic rpL5 peptide variants. The data for the wild-type N130+rpL5 system are replotted from Fig. 1e. (b) *g*_OD-OD_(*r*) between the ODs for dense phases of each synthetic peptide + N130 mixture. The disordered linker weakens nearest neighbor interactions whilst preserving the strengths of and shifting the preferred positions of next-nearest-neighbor correlations. Averages were calculated over five replicates. The sequences of the linkers are as follows: 10L: GSRRRRGSGSYYGSGSRKRLV; 16L: GSRRRRGSGSGSGYYSGSGSGSRKRLV; and 20L: GSRRRRGSGSGSGSGYYSGSGSGSGSRKRLV.

Next, we investigated the effects of rpL5 on network fluid structure. We expected that changes to the effective lengths of crosslinkers will impact the length scales and the magnitudes of the spatial inhomogeneities. To investigate this, we created synthetic variants of rpL5 in which the residues between the two basic tracts were replaced by synthetic linkers comprising 10, 16 or 20 residues (termed 10L, 16L and 20L, respectively). First, we obtained SANS data for N130 complexed with the three different synthetic rpL5 peptides (Fig. 5a). The differences in the low-*q* regime, where *q* < 0.03 Å^−1^, can be attributed to the different form factors or shapes of the peptides rather than to differences the structure of the dense phase. In the intermediate *q*-regime, the correlation peaks shift to lower *q*-values upon increasing the length of the peptide. These peaks, which correspond to the nearest neighbors, indicate that the neighborhood is extended by the longer linkers wherein the GS repeats impart flexibility to the linkers.

To gain insights into the structural effects of the synthetic rpL5 peptides, we performed simulations for dense phase facsimiles of N130 complexed with each of the three peptides and computed the corresponding *g*_OD-OD_(*r*) profiles (Fig. 5b). The reduced height of the first peak points to a disruption of nearest neighbors by the peptides with longer linkers (eight residues link the two basic motifs in the rpL5 peptide). However, the prominence of the second peak is maintained although the positions are shifted toward larger separations. Maintenance of the second peak highlights the fact that this peak is mainly due to interactions between R-motifs and acidic tracts.

Taken together, the results show that N130+rpL5 condensates are network fluids, reminiscent of associative liquids such as water and patchy colloids, as opposed to simple liquids, where structure is defined purely by the packing of spherical particles. Further, by titrating the strengths or contributions of acidic regions on N130 and the linear charge density of R-motifs in rpL5, we find that charge neutralization through complexation plays an important role in the intermediate and long-range structural features. The linear charge density, influenced by extending the linkers between R-motifs, alters the short-range ordering of ODs.

### Graph theoretic analyses of network structures of condensates

To put the network fluid concept on a quantitative footing, we turned to graph-theoretic approaches. This is helpful because it overcomes weaknesses inherent to analyses of the structure factors and radial distribution functions, which are one-dimensional projections that average over time and molecular orientations. Graph-theoretic approaches have been helpful in analyzing network fluids, for example in studies of hydrogen-bonding networks^95, 96, 97, 98, 99, 100^ and glassy systems^101^. Here, we perform a graph-theoretic analysis of the macromolecular network formed by the N130+rpL5 dense phase in the CG simulations.

**Fig. 6:**
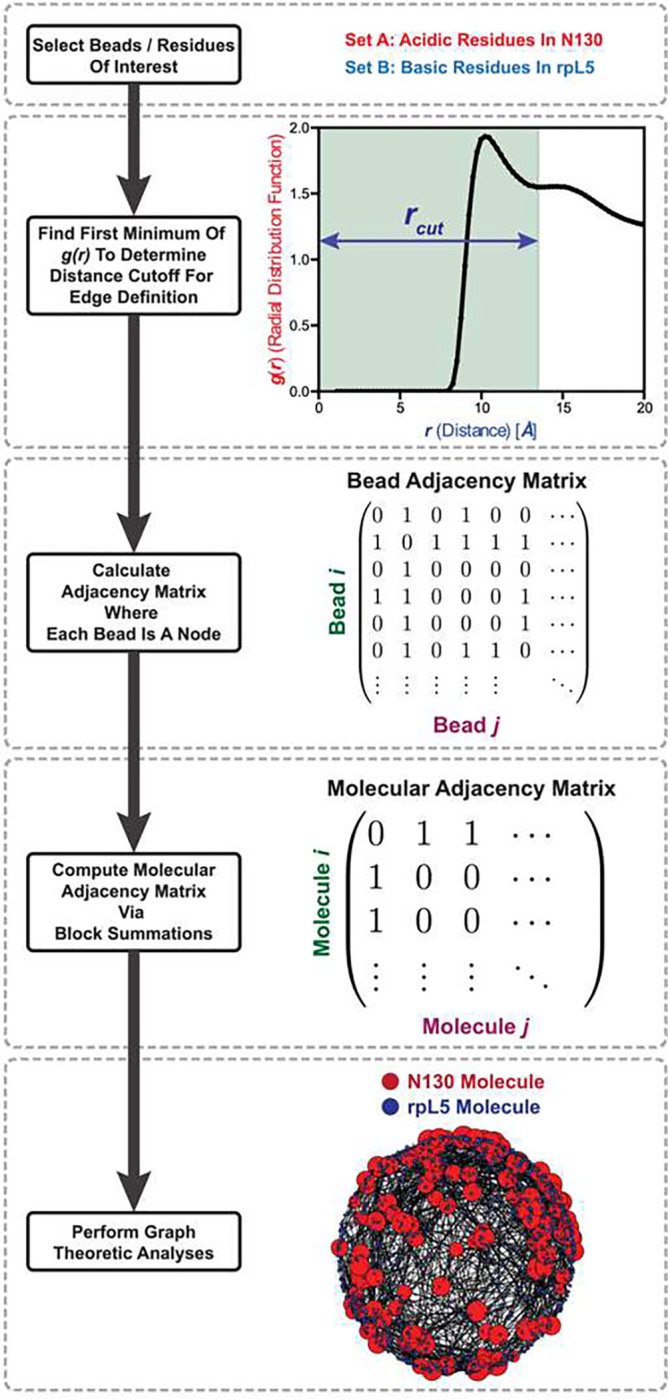
Flowchart describing the graph-theoretic analysis of coarse-grained simulations of dense-phases of N130 and rpL5. We start by selecting a set of residues of interest. As an illustrative example, we pick the acidic residues in N130 and the basic residues in rpL5. To determine an edge, we compute the g(r) between sets of beads where the first minimum serves as the distance cutoff for bead adjacency. Given this cutoff, we construct the bead-to-bead adjacency matrices. The system includes the multidomain N130, and performing appropriate block-summations of the bead-to-bead adjacency matrix generates the molecular adjacency matrix. The latter matrix is analyzed using standard graph-theoretic analyses. As an example, a random embedding is shown where the size of a node corresponds to the degree centrality of that node.

Following previous work on molecular fluids ^102^, we construct unweighted graphs in which two molecules are considered adjacent if any of the constituent beads are within the cutoff distance defined by the first minimum in the corresponding g(r). Using this criterion, we constructed appropriate adjacency matrices via block summations (Fig. 6). We then analyzed the network structure formed by the molecular neighbors for the set of beads considered.

**Fig. 7:**
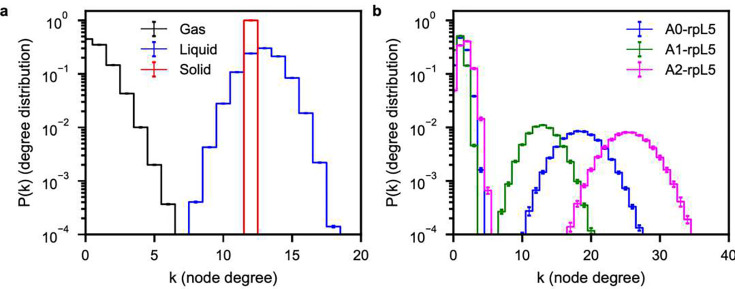
Each acidic region of N130 in the N130+rpL5 dense phase imparts a different network structure onto the system. **(a)** Degree distributions, P(k), for pure Lennard-Jones (LJ) systems. Because the density of the vapor (LJ) is low, the most likely degree is zero. For the liquid ρ=0.01,T=1.000), the degree distributions shift towards higher values with a mean around twelve, where the widths of the distributions correspond to the inherent variation in the number of bonds particles can make in the locally spatially inhomogeneous environment of a liquid. For the solid (ρ=1.5,T=0.758), the degree distribution peaks very sharply at twelve. **(b)** Degree distributions, P(k), for the complementary charge interactions between the different acidic tracts of N130 and the rpL5 peptides. Unlike the graphs in panel (a), the distributions display bimodality, which is an indication of a bipartite graph. Consistent with the radial distribution functions in Fig 3, we see that A2 has the largest degrees, followed by A0 and A1. In all cases, averages were calculated across five replicates.

To provide a suitable prior of a non-networked fluid where structure is dominated by packing considerations alone, we performed graph-theoretic analyses on the Lennard-Jones system. For this, we quantified the degree distributions for the vapor, liquid, and solid phases of spherical particles interacting via Lennard-Jones potentials (Fig. 7a). In graph theory, the degree reflects the number of connections or edges emanating from a node. Here, a node is an individual Lennard-Jones particle. For the pure vapor, the most likely degree is zero, and this is in accord with expectations for an ideal gas. However, since Lennard-Jones particles have finite size and there are attractive dispersion interactions, the vapor phase is not ideal. Instead, the degree distribution is skewed to the right. For the Lennard-Jones liquid, we observe a broad distribution that is approximately symmetric about a degree value of 13. As the density is further increased to obtain a solid, we see that the degree distribution shows a sharp peak at twelve, corresponding to the number of neighbors expected for a 3D hexagonal close-packed lattice ^103^. Note that the locally inhomogeneous nature of a liquid allows for interactions with more neighbors than the true ground-state number seen in the solid phase.

Next, we constructed graphs using acidic residues in N130 and the basic residues in rpL5 as nodes. In contrast to the Lennard-Jones systems, the computed degree distributions are bimodal (Fig. 7b), and this is suggestive of a bipartite network structure. The multimeric nature of the N130 pentamer allows the acidic regions to interact with multiple rpL5 peptides, as seen in the broad second peaks in the degree distributions. Consistent with the radial distribution functions for N130+rpL5, we also observe a hierarchy of degrees, with A2-rpL5 having the largest degree and A1-rpL5 having the smallest. However, for the first peaks near *k* = 0, which correspond to the smaller rpL5, we see that the different acidic regions do not show appreciable differences. Comparison to the Lennard-Jones system suggests that the N130+rpL5 system features both liquid- and gas-like interactions. The gas-liquid behavior points to presence of unassociated, freely diffusing rpL5 coexisting with the associated rpL5 molecules.

In spatially inhomogeneous systems, there can be regions that are locally dense or dilute. This is made clear in the graph-theoretic analysis, which shows two interaction modalities. Similar results have been reported for fluids formed by patchy particles, especially near the liquid-gas coexistence region ^79, 80, 81^. The totality of our structural analyses suggests that N130+rpL5 condensates are akin to network fluids formed by patchy and / or hairy colloidal particles. The coexistence of liquid- and gas-like organization within the condensates gives rise to dynamical consequences that we discuss next.

### Dynamics within network fluids show two distinct regimes

**Fig. 8:**
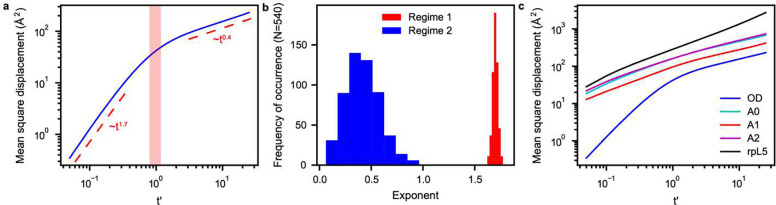
Motions within dense phases of N130+rpL5 show bimodality. **(a)** MSD of the OD plotted against the lag time shows that N130 has super-diffusive and sub-diffusive regimes. Here, the abscissa is a unitless parameter $t^{\prime }=t/t_{D}$ where $t_{D}$ is the timescale over which the motion of the OD is purely diffusive. The red region in the panel indicates the timescales that fit best to purely diffusive motion. There is a timescale below $t_{D}$ where the motion is super-diffusive and a timescale above $t_{D}$ where the motion is sub-diffusive, with the regimes and corresponding exponents indicated in the panel. **(b)** Histograms of the exponents calculated for the mean square displacements of individual ODs. The bimodal distribution reflects the presence of super-diffusive and sub-diffusive regimes. **(c)** MSDs of the OD and charged residues in each of the acidic regions and the rpL5 peptides. In contrast to the OD, the acidic regions and the peptides show only sub-diffusive motions on all timescales. In all cases, averages were calculated over five replicates.

We analyzed the simulations to compute mean square displacements (MSDs) of the ODs. The MSD is calculated as a function of lag time. This involves a double average, where the inner average is a cumulative sum along the time axis, starting from zero, and progressing in increments of t+Δ, where the mean square displacement is computed over times t and t+Δ and averaged over the motions of individual molecules. The outer average is over all molecules. A characteristic timescale corresponds to the time it takes for the OD to diffuse across a distance corresponding to its diameter. Since the simulations are solvent-free and involve coarse-grained descriptions of molecules, we rescaled the abscissa by $t_{D}$, which is the timescale over which the motion of the OD fits best to a purely diffusive model with MSD being proportional to t. We find that there is a timescale below $t_{D}$ where the motion is super-diffusive with an exponent greater than one, and a timescale above $t_{D}$ where the motion of the OD is sub-diffusive with an exponent less than one (Fig. 8a). Based on the observed length scales, the super-diffusive motion reflects the short-range steric repulsions among the ODs and the electrostatic repulsions between acidic residues. Histograms of the exponents that we compute for the MSDs show a bimodal distribution (Fig. 8b). The distribution of sub-diffusive exponents is broader and reflects the heterogeneities of motions impacted by associative interactions between acidic regions and rpL5 peptides. The MSDs calculated for the OD and for charged residues in each of the acidic regions and the basic residues in the rpL5 peptides contrast with the MSDs computed in terms of the OD alone (Fig. 8c). The acidic regions and the peptides show sub-diffusive motions on all timescales, reflecting the fact that these moieties are influenced mainly by associative intermolecular interactions.

The sub-diffusive dynamics derived from simulations are consistent with the apparent plateau in the FRAP data for the wild type (Fig. 2d) at long timescales. Similar retarded motions of NPM1 were observed in FRAP analysis of condensates formed by NPM1 and a R-rich N-terminal region (S6N) of the nucleolar protein SURF6 ^8^. Significant fractions of NPM1 molecules were found to be immobile at all S6N:NPM1 ratios. Further, using nuclear magnetic resonance measurements, Gibbs et al., found that the ODs of NPM1 form an immobilized scaffold in NPM1+p14ARF mixtures ^104^. Super- and sub-diffusive behavior of the core particles was also observed in MSDs computed from simulations of oligomer-grafted nanoparticles ^105^.

## Discussion

Condensates have been described as structureless liquids defined by non-specific interactions ^106^. However, even simple liquids, a term reserved for Van der Waals liquids whose structures are defined purely by packing effects ^92^, will exhibit short-range order and long-range disorder. Importantly, liquid-state structures are directly connected to the molecular architectures, the spatial range, types, and strengths of intermolecular interactions ^93^. Further, the extent and type of ordering is enhanced in fluids with architectures that enable anisotropic interactions such as hydrogen bonding. Since condensates do not arise purely from segregative transitions such as LLPS, one should expect there to be spatial inhomogeneities, and a network-like organization that reflects the physical, gel-like characteristics engendered by associative macromolecules ^22^.

Here, we deployed a combination of experimental and computational techniques to demonstrate that condensates formed by N130 and rpL5 are network fluids. This was established by observing correlation peaks in SANS curves of condensates that are indicative of molecular order on the length scale of the N130 OD. These data were bolstered by comparative computational assessments of the radial distribution functions to Lennard-Jones fluids, which are simple liquids as opposed to associative fluids. The condensates formed by N130 and rpL5 show fluid-like organization indicated by short- and intermediate-range ordering, and long-range disorder. The short- and intermediate-range ordering can be explained in terms of an encoded hierarchy of interactions involving the disordered acidic regions of N130 and the R-rich motifs of rpL5.

The sequence-specific CG model allowed us to identify a new acidic region, termed A0, in the N-terminal OD of NPM1. We find that A0 interacts more strongly with the disordered peptide rpL5 than was hitherto appreciated. Mutations that increase the charge in A0 help lower the threshold concentration of rpL5 that is needed to observe condensation driven by heterotypic interactions with N130. Further, we find that increasing the charge in the A0 region slows the dynamics within N130+rpL5 condensates, as measured by FRAP.

The layering in terms of short- vs. intermediate-range ordering versus long-range disorder that is observed in the SANS data can be reproduced via a bespoke coarse-grained model, which was used to compute radial distribution functions. From the computed g(r) profiles, we find that N130 has four nearest neighbors on average. This is reminiscent of the coordination statistics for liquid water, which is the most well-known small-molecule network fluid. Complexation between the acidic tracts and the rpL5 peptides also contributes to the overall structure of the condensates. Interestingly, the acidic regions function as independent modules, and this explains why the valence of such motifs is shown to be an important driver of condensation and the measured material properties ^63^.

In accord with findings for patchy colloids, we find that there are two types of sub-graphs that underlie the structure of the N130+rpL5 condensates. One of the sub-graphs corresponds to gas-like organization, and the other corresponds to that of a liquid. Note that the term “gas-like” implies that there are regions within condensates where the concentrations of macromolecules are ultra-dilute, and hence solvent filled. This is akin to the empty liquid concept ^107^ that has emerged from the patchy colloid literature. Conversely, what we refer to as “liquid-like” refers to regions that are dense in macromolecules and hence likely to be deficient in solvent. Importantly, the bipartite graphs also have dynamical fingerprints, which manifest as the bimodality we observe for the MSDs of the ODs. Overall, our findings place the N130+rpL5 system, and presumably other such systems, in the same category as patchy and / or hairy colloids ^79, 80, 81, 83, 105, 107, 108, 109^.

In this work, we focused mainly on the effects of heterotypic interactions between N130 and R-rich rpL5 peptides on the network structure of the N130+rpL5 condensates. Previous work has shown that the homotypic interactions within NPM1 can also affect both the phase behavior and the mesoscopic structure of condensates formed with SURF6N ^8, 64^. An application of graph-theoretic analysis, guided by SANS measurements, to condensates that form mainly under the influence of homotypic interactions should be feasible through the combination of methods deployed in this work. The interplay between network structures defined by the whole range of homotypic and heterotypic interactions should illuminate the relationship between the rheological properties and network structure – as has been shown recently for a system of prion-like low complexity domains ^50^.

Since the nucleolus is a multicomponent and multiphasic condensate, we expect that varying the stoichiometries of the different components will affect the overall structural properties of nucleoli. Future studies that apply the combination of experimental, computational, and analytical techniques deployed here to more complex systems will enrich our understanding of the relationship between the spatial organization of condensed systems and the network properties.

## METHODS

### Cloning

All NPM1-derived N130 constructs were subcloned into a pET28b plasmid vector, in frame with an N-terminal 6x His tag, followed by a TEV protease recognition sequence. rpL5 peptides with variable linkers (rpL5–10L, rpL5–16L, rpL5–20L) were subcloned into a pET28b vector, in frame with an N-terminal 6x His tag and the GB1 protein ^110^, followed by a TEV protease recognition sequence.

### Protein expression and purification

The plasmid constructs were used to transform in *E. coli* strain BL21(DE3) (Millipore Sigma, Burlington, MA, USA), followed by incubation with shaking at 37 °C. When bacterial cultures reached an optical density at 600 nm of ~0.8, the temperature was reduced to 20 °C and protein expression was induced with the addition of IPTG (GoldBio, St. Louis, MO, USA) to a final concentration of 1 mM, and further incubated with shaking overnight. Cells were harvested by centrifugation and lysed by sonication in buffer A (25 mM Tris, 300 mM NaCl, 5 mM β-mercaptoethanol, pH 7.5). The soluble fraction was further separated by centrifugation for 30 min at 30,000 x g and loaded on a Ni-NTA column, pre-equilibrated in buffer A. Bound protein was eluted with a gradient of buffer B (25 mM Tris, 300 mM NaCl, 500 mM Imidazole, 5 mM β-mercaptoethanol, pH 7.5). The fractions containing the protein of interest were identified by SDS-PAGE, pooled and the 6x His affinity tag was removed by proteolytic cleavage, in the presence of TEV protease, while dialyzing against 4 L of 10 mM Tris, 200 mM NaCl, 5 mM β mercaptoethanol, pH 7.5. To remove the cleaved affinity tag and any un-cleaved material, the protein was applied to an orthogonal Ni-NTA column, and the flow-through loaded on a C4 HPLC column, in 0.1% Trifluoroacetic acid, and eluted with a linear gradient of 0.1 % Trifluoroacetic acid in acetonitrile. The fractions containing the proteins of interest were identified by SDS-PAGE, pooled and lyophilized. Lyophilized N130 and N130^+A2^ proteins were resuspended in 6 M Guanidine hydrochloride, 25 mM Tris, pH 7.5 and reduced by the addition of 10 mM dithiothreitol. The proteins were refolded by dialysis, using 3 exchanges of 10 mM Tris, 150 mM NaCl, 2 mM mM dithiothreitol, pH 7.5, at 4 °C. The protein concentration during refolding was maintained at or below 100 μM N130 monomer. Protein identities were verified by determining their molecular weight using mass spectrometry in the Center for Proteomics and Metabolomics at St. Jude Children’s Research Hospital.

### Fluorescence labeling of proteins

N130 and N130^+A2^ were labeled with Alexa-488 (ThermoFisher, Waltham, MA, USA) at Cys104 by incubating a molar excess of Alexa-488 maleimide with freshly reduced N130 proteins overnight at 4 ℃ with oscillation. Excess dye was removed by successive rounds of dialysis against 10 mM Tris, pH 7.5, 150 mM NaCl, and 2 mM DTT. Labeled proteins were then unfolded in the presence of 10 mM Tris, pH 7.5, 2 mM DTT, and 6 M GdmHCl and combined with unlabeled protein to a final concentration of 10% labeled protein and refolded by successive rounds of dialysis against 10 mM Tris, pH 7.5, 150 mM NaCl, and 2 mM DTT.

### Fluorescence microscopy

Microscopy plates (Greiner Bio, Kremsmünster, Austria) and slides (Grace BioLabs, Bend, OR, USA) were coated with PlusOne Repel Silane ES (GE Healthcare, Pittsburgh, PA, USA) and Pluronic F-127 (Sigma-Aldrich, St. Louis, MO, USA) and washed with water before the transfer of protein solutions. Fluorescent microscopy experiments were performed using a 3i Marianas spinning disk confocal microscope (Intelligent Imaging Innovations Inc., Denver, CO, USA) with a 100X oil immersion objective (N.A. 1.4). The phase diagram depicted in Figure 2d was generated by computing the average of the index of dispersion of fluorescent microscopy images of 5 images per well. The threshold for positive phase separation has been set to 10% of the maximum value. Fluorescent recovery after photobleaching (FRAP) experiments were performed by bleaching a circular area with a diameter of 1 μm in the center of droplets (n=12) to approximately 50% of initial fluorescence intensity. The observed fluorescence intensities were then normalized to global photobleaching during data acquisition and fitted as a group to determine recovery times according to ^111^

It=I0+I∞tt121+tt12

Uncertainty in the reported half-lives represent the standard error of the fit data. FRAP experiments were performed one hour after the mixing of components.

### Peptides

The rpL5 peptide was synthesized in the Macromolecular Synthesis lab at the Hartwell Center, St. Jude Children’s Research Hospital. Peptides with variable linkers were recombinantly expressed in *E. Coli* BL21(DE3) (Millipore Sigma, Burlington, MA, USA), and purified from the soluble fraction as described above. The lyophilized powder was directly reconstituted in buffer, and the pH was adjusted to 7.5 using 1 M Tris base.

### Small-angle neutron scattering

N130 and N130^+A2^ were buffer exchanged into 10 mM Tris, 150 mM NaCl, 2 mM DTT, in D_2_O (measured pH, 7.5). Lyophilized rpL5 peptides were resuspended in dialysis buffer. Monodisperse samples of protein only and phase separated samples with rpL5 were prepared in the dialysis buffer. SANS experiments were performed on the extended Q-range small-angle neutron scattering (EQ-SANS, BL-6) beam line at the Spallation Neutron Source (SNS) located at Oak Ridge National Laboratory (ORNL). In 30 Hz operation mode, a 4 m sample-to-detector distance with 2.5–6.1 and 9.8–13.4 Å wavelength bands was used ^112^ covering a combined scattering vector range of 0.006 < *q* < 0.44 Å^−1^. *q* = 4π sin(θ)/λ, where 2θ is the scattering angle and λ is the neutron wavelength. Samples were loaded into 1 or 2 mm pathlength circular-shaped quartz cuvettes (Hellma USA, Plainville, NY, USA) and sealed. SANS measurements were performed at 25 °C using the EQ-SANS rotating tumbler sample environment to counteract condensate settling. Data reduction followed standard procedures using MantidPlot^113^ and drtsans.^114^ The measured scattering intensity was corrected for the detector sensitivity and scattering contribution from the solvent and empty cells, and then placed on absolute scale using a calibrated standard ^115^.

### Atomistic Monte Carlo simulations using the ABSINTH model

For the first step of systematic coarse-graining, we performed atomistic Monte Carlo simulations to obtain a robust description of the conformational ensembles of N130. For this, we employed the ABSINTH implicit solvation model and forcefield paradigm ^77^. In this model, all polypeptide atoms and solution are modeled explicitly, and the degrees of freedom are the backbone and sidechain dihedral angles as well as the translational motions of the solution ions, which are spheres. All simulations were performed using version 2.0 of the CAMPARI modeling package (http://campari.sourceforge.net) and the abs_opls_3.2.prm parameter set. The initial structure of N130 was modelled as a pentamer, where the structure of the oligomerization domains is based on the coordinates deposited in the protein data bank (PDB ID: 4N8M). The structures of each disordered N-terminal tail (residues 1–18, GSHMEDSMDMDMSPL) and disordered A2 tract (residues 124–133, EDAESEDEDE) were built using CAMPARI. The degrees of freedom internal to the oligomerization domains were held fixed during the ABSINTH simulations, reflecting the fact that the domains are well folded and tightly bound to each other. The system is placed in a soft-wall spherical potential with radius 70 Å. We included sodium and chloride ions to mimic the salt concentration of ~20 mM, in addition to neutralizing ions. The simulation temperature was set to 300 K.

For efficient sampling of the conformational ensemble, we first performed simulations based on the so-called excluded volume or EV limit. In this limit, all terms other than the steric repulsions and any dihedral angle terms in the potential functions are switched off. These initializing simulations were performed for 10^8^ Monte Carlo (MC) steps. We sampled 100 different structures from the EV limit simulations and used each of them as initial structures simulations based on the full potential. Each simulation consists of 10^8^ MC steps, and the structural information was stored every 5,000 steps. Hence, we collected 20,000 snapshots per trajectory, from 100 independent trajectories of atomistic simulations.

For each of the rpL5 peptide and its variants, we generated 50 initial structures using build routines in CAMPARI. Each system was then placed in a soft-wall spherical potential with radius 45 Å, along with sodium and chloride ions to mimic the salt concentration of ~20 mM. The simulation temperature was set to 300 K. We performed ABSINTH-based MC simulations for 2.1×10^7^ MC steps with sampling frequency of (5,000 steps)^−1^, where the first 10^6^ MC steps were discarded as equilibration.

### Systematic coarse-graining

Our approach to developing bespoke coarse-grained models involves three key steps: First, we choose the resolution for the coarse-grained (CG) model. Second, we choose the form for the potential functions that describe interactions among pairs of coarse-grained sites. And third, we use a combination Gaussian process Bayesian optimization (GPBO) module to parameterize the model to ensure that the CG model recaptures conformational statistics of the atomistic simulations. In our choice of the model, each residue is modelled as a bead, except for the oligomerization domain of N130, which by itself forms a large bead with excluded volume. The mass of each bead is determined by the total mass belonging to the specific bead. For example, the oligomerization domain bead has mass of 47544.8 amu. The position of each bead is set equal to the position of the center of mass in its atomistic representation.

The potential function used for the simulations is decomposed into five different terms:

(1) $$W=W_{LJ}+W_{el}+W_{b}+W_{\theta }+W_{\phi }$$

Each term contains several interaction parameters, which were parameterized using the CAMELOT algorithm. This uses a GPBO module to minimize an objective function, which is defined as the difference between the residue-residue distributions generated by atomistic simulations and by coarse-grained simulations. CAMELOT iteratively changes the parameters of the potential function for the CG model, performs CG simulations to obtain conformational statistics, specifically inter-bead distance distributions, and iterates this process until a stationary state is reached for the objective function. In this work, we used conformational statistics derived from the atomistic simulations of structural ensembles of N130 and R-rich peptides as the reference against which the CG model was parameterized. The optimized parameters are given in Supplementary Tables 1–23.

To reduce the computational cost of scanning the parameter space, we grouped several amino acid types into one bead type, following the previous work. For the rpL5 peptide and its variants, we grouped different amino acids into three different bead types: *charged* (K, R, E, D), *large* (V, F, M, I, L, Y, Q, N, W, H), and *small* (A, P, G, S, T, C). Each group has its own $\epsilon_{i}$ value to be determined. For each residue in either the large or small group, we used $\sigma_{i}$ as twice of the radius of gyration of the specific residue in the atomistic simulations (and consequently $\sigma_{i}$ is not identical for all residues in the same group; it is residue-dependent). For charged residues, its $\sigma_{i}$ was left as a free parameter to determine by the optimization module. Hence, we have four unknown parameters, three $\epsilon_{i}$ values for the charged, large, and small bead types plus one $\sigma_{i}$ value for the charged bead type

### Coarse-grained model

The coarse-grained (CG) model is summarized in Fig. 1, and the potential function used for the simulations can be decomposed into five different terms given in Eq. 1. Here,

$$W_{LJ,ij}=4\epsilon_{ij}\left[{\left(\frac{\sigma_{ij}}{r_{ij}}\right)}^{12}-{\left(\frac{\sigma_{ij}}{r_{ij}}\right)}^{6}\right],\;r_{ij}<r_{c}$$

is the standard LJ potential with cutoff $r_{c}=2.5\sigma$. While we decomposed the two-body interaction parameters into one-body parameters: $\sigma_{ij}=\left(\sigma_{i}+\sigma_{j}\right)/2$ and $\epsilon_{ij}=\sqrt{\epsilon_{i}\epsilon_{j}}$. All energies have units of kcal / mol.

The electrostatic interactions were modeled using a Debye-Hückel potential given by

$$W_{el,ij}=C\frac{q_{i}q_{j}}{\epsilon r_{ij}}exp\left(-\kappa \;r_{ij}\right),\;r_{ij}<r_{c}$$

implemented as the `lj/cut/coul/debyè pair-style in LAMMPS ^116^ with $\epsilon =80.0,\;\kappa =0.1,\;\sigma_{1}=24.1$ and $\sigma_{2}=15.0\;\text{Å}$. with constant C, charges q, dielectric constant ϵ, and inverse Debye length κ. In this work, we used ϵ=80.0 and $\kappa =0.1\;{\text{Å}}^{-1}$. The charges were assigned manually; beads for R and K have +1, beads for D and E have −1, and other beads (including the OD bead) have 0.

The bond and angle terms are modeled as harmonic potentials,

$$W_{b,i}=K_{i}{\left(b_{i}-b_{0i}\right)}^{2}$$

is a quadratic bonded potential implemented as the harmonic bond-style in LAMMPS ^116^.

$$W_{\theta ,i}=K_{i}{\left(\theta_{i}-\theta_{0i}\right)}^{2}$$

is a quadratic angular term implemented as the harmonic angle-style in LAMMPS ^116^. The bond parameters $K_{b,i}$ and $b_{0i}$ were obtained by fitting the normal distribution to the distribution of the distance between two adjacent residues. The angle parameters $K_{a,i}$ and $\theta_{0i}$ were also obtained by fitting the normal distribution to the distribution of the angle between three adjacent residues. For the oligomerization domain bead, we assigned arbitrarily high values for the energy parameters: $K_{b,i}=60000\;kcal/mol-{\text{Å}}^{2}$ and $K_{a,i}=\;6000000\;kcal\;/\;{mol-radian}^{2}$, keeping the bond extremely rigid. The equilibrium length and angle were determined by the PDB structure.

Except for the oligomerization domain bead, the dihedral term is given by a Fourier series potential,

$$W_{\phi ,i}=\sum_{n=1}^{3}K_{ni}\left(1-cos\left(n\phi_{0i}-\phi_{ni}\right)\right)$$

is a Fourier series dihedral term implemented as part of the class2 dihedral style in LAMMPS. with arbitrarily high value of $K_{d,i}=\;6000000\;kcal\;/\;{mol-radian}^{2}\;$ and experimentally determined $\phi_{0i}$. The parameters corresponding to the potentials, derived from CAMELOT, for the different systems considered are in SI Tables 1–23. Lastly, $W_{\phi ,i,quad}=K_{i}{\left(\phi_{i}-\phi_{0i}\right)}^{2}$ is used to constrain the five arms of N130 with a very high K -values.

### Lennard-Jones fluids

Using the `lj/melt` example in the LAMMPS documentation as a starting point, we performed standard NVT simulations, with 10^4^ Lennard-Jones particles, to understand the network structure of different LJ phases. To access different pure phases, we used the NIST parameters for a pure LJ gas, a pure LJ fluid, and a pure LJ solid. In LJ units, the densities and temperatures for the different systems are in SI Table 1.

With the standard dt=0.005τ, where τ is the dimensionless Lennard-Jones time unit, an initial $t_{\text{therm}}=1\times 10^{5}$ timesteps were run to let the systems settle, after which for $t=1\times 10^{7}$ timesteps were run for data production. Trajectory snapshots were output every 10000 timesteps. The last 500 frames were used to calculate the radial distribution functions (RDFs) and degree distributions. Three independent replicates were run for each condition, and the standard error of the mean between replicates is used as the measure of uncertainty.

### Simulating of the N130 wild type and rpL5 peptide variants

Given the initial configuration generated using CAMELOT ^76^, we used the command `replicate 3 3 4` to generate 3 × 3 × 4 = 108 copies of N130 and 15 × 108 = 1620 of the rpL5 peptide. Following the replication, `fix deform` was used with `fix nve/limit` to reduce the box-sizes for the simulations to 250 nm. The final configuration served as initial conditions for NPT simulations to prepare systems at the correct intrinsic density. NPT simulations were run for $t=1\times 10^{7}$ timesteps. The final configurations from these NPT simulations served as the starting configuration for NVT simulations which were run for $t=1\times 10^{9}$ timesteps. Trajectory snapshots were output every 50000 steps, and we only considered the last 1000 frames for the different analyses. Five independent NVT replicates were run for each condition, and the standard error of the mean between replicates is used as the measure of uncertainty.

### Simulations of the N130 mutants

Using the final box-size from the NPT simulations with N130 and the native rpL5 peptide, we ran NVT simulations for $t=2\times 10^{9}$ timesteps. Like the peptide variant simulations, trajectory snapshots were output every 50000 timesteps in the last 1 × 10^G^ steps, and only the last 1000 frames were considered for analyses. Five independent NVT replicates were run for each condition, and the standard error of the mean between replicates is used as the measure of uncertainty.

### Calculation of the radial distribution functions g(r)

We used VMD^117^ to calculate g(r) for the different sets of beads considered in this work. The specific function used was `measure gofr`. To calculate the g(r) for the N130+rpL5 system, we used a bin size, dr, of 0.5 Å. Note that the g(r) profiles for the N130 mutants were computed from the pair distributions between all the acidic residues in the select acidic tract, and all basic residues in the peptide. For the LJ-fluid simulations, we used a bin size of 0.025 in units of dr/σ. As with our analysis of the N130+rpL5 system, we averaged over all replicates.

### Graph-building methods

First, we decide which part of the network structure of the fluid we wish to investigate. In the example shown in Fig. 6, we pick the network formed by the acidic residues in a particular acidic tract in N130 and the basic residues in rpL5. Then, given these two sets of residues / beads, we calculate the inter-set g(r) as explained in the methods. Given this g(r), we find the location of the first minima from each of the computed g(r) profiles. The locations of these minima serve as the cut-off radii for defining the presence of an edge between beads.

Given the two sets of beads, a particular trajectory snapshot, and the computed cut-off radius, we generate a bead-adjacency matrix. As an example, we can pick a specific acidic residue from N130. A basic bead is considered adjacent if the distance between the selected acidic bead and the basic bead is within the cutoff radius. This calculation is performed for all pairs defined by the two sets initially chosen. This generates a bead adjacency matrix where an edge is drawn between those beads where the inter-bead distances are within the computed cutoff radius. We can either consider the total bead adjacency matrix where every bead in the system is included and where all the bead-types not in the initially chose set are non-adjacent by construction, or we generate a bead adjacency matrix where only the considered beads are included. We choose the latter.

In more detail, suppose that set-1 has m -bead *types* and set-2 has n-bead *types*. Then, given α N130 molecules, set-1 has α⋅m beads in total. Similarly, given β rpL5 molecules, set-2 has (β⋅n) beads in total. Therefore, the bead-adjacency matrix will be an (α⋅m+β⋅n)×(α⋅m+β⋅n) matrix. As an example, suppose we have 1 N130 molecule, and 1 rpl5 molecule. This would then give us an (m+15n)×(m+15n) matrix. Furthermore, suppose that the beads are ordered such that the first m -rows correspond to the N130 beads, and so the next n-rows are the rpL5 beads (since adjacency matrices are symmetric the first m-columns would be for N130 and the next n-columns would be for rpL5). To go from this bead adjacency matrix to a molecular adjacency matrix we would look at blocks of this bead adjacency matrix. Let the bead-adjacency matrix be $\overset{ˆ}{B}$. Using Matlab style indexing, B[0:m, 0:m] corresponds to the sub-graph of N130 adjacent beads. In our case, no beads will be adjacent since the graph is intentionally constructed between the acidic residues in N130 and the basic residues in rpL5. Moving on, B[0:m, m:m+n] (or B[m:m+n, 0:m] due to symmetry) corresponds to the sub-graph between the N130 beads and the beads of the first rpL5 molecule. Similarly, if we had more than 1 rpL5 molecule, B[0:m, m+i*n:m+(i+1)*n] gives us the subgraph between the N130 beads and the beads of the i-th rpL5 molecule. To generate the molecular adjacency matrix, $\overset{ˆ}{A}$ we check if any of the sub-graphs from the bead-adjacency matrix are non-empty (or that there are edges in that graph) or that there is a 1 in the B[0:m, m+i*n:m+(i+1)*n] block. Therefore, A[0,0]=0 in our case by construction, or that molecule-0 and molecule-0 are *not* adjacent.

Returning to the more general case, we have the bead adjacency matrix that corresponds to a matrix of size (α⋅m+β⋅n)×(α⋅m+β⋅n) where the first α⋅m rows (or columns) correspond to the beads in N130 molecules, and the last β⋅n rows (or columns) correspond to the beads in rpL5 molecules. We check all the blocks of the matrix which correspond to the beads between the different molecules in the system. These correspond to the subgraphs between the beads of different molecules. For any sub-graph or block that is non-empty, we consider the two corresponding molecules to be adjacent. This then gives us the molecular adjacency matrix which should have the shape (α+β)×(α+β). This is the final graph that is then analyzed. Graph properties are calculated per-snapshot and are then averaged over the total set of frames considered.

This molecular adjacency graph is constructed by considering the adjacency between any of the beads from the initially selected set. Since we only care about the acidic and basic beads, by construction this graph avoids self-loops. Furthermore, since we only care about specific blocks of the bead adjacency matrix being non-empty, we only get an unweighted graph. If, however, we wanted to get the weighted graph, we would simply take the sum of the number of edges in a particular sub-graph or take the sum of that block from the bead adjacency matrix.

On an implementation level, we can skip most of the block reductions etc. by simply asking first: given the two sets of beads and the cutoff radius, which pairs of beads are adjacent. Then, from the pairs of adjacent beads, we ask which molecules the beads in the pairs come from. More specifically we ask what the molecule-ID of each bead is. From this set of molecule-ID pairs, we simply find the unique pairs. Given these unique pairs of adjacent molecule-ID’s, we cane explicitly construct the molecular adjacency matrix since the molecule-ID’s directly correspond to the indices in the adjacency matrix. We set those elements of the (α+β)×(α+β) matrix to 1, and we get our molecular adjacency matrix.

### Degree distributions

From a given trajectory snapshot, we generate molecular graphs, G(V,E), where individual molecules are represented as nodes, V. To calculate the edges between nodes in a generalizable way, we use the first minima from the RDFs of the given sets of beads. We use signal.find_peaks from the SciPy package^118^ to find these minima. We generated RDFs for the acidic / negatively charged beads in N130 from a particular acidic tract, and the basic residues in the rpL5 peptide. Given the RDFs, we then use the first minimum as the cut-off radius for the definition of an edge. An edge, E, is therefore drawn between two nodes if any of the beads from the considered sets are within the distance corresponding to the first minima of g(r). We use MDAnalysis^119^ to analyze the trajectories to find adjacent molecules. Given these adjacency matrices, we then calculate the degree of each node by calculating the total number of edges for each node, or by calculating the sum of each row (or column) of the adjacency matrix. The python package NumPy^120^ is used to generate these degrees. Given these degrees, we calculate the degree distribution. The degree distributions from the last 1000 frames are averaged over, and the average over the five simulation replicates are reported here.

### Mean square displacements

The mean square displacement (MSD) was calculated by averaging the displacements in particle positions over all windows of length m and over all particles N:

(2) $$MSD(m)=\frac{1}{N}\sum_{i=1}^{N}\frac{1}{N-m}\sum_{k=0}^{N-m-1}{\left({\vec{r}}_{i}(k+m)-{\vec{r}}_{i}(k)\right)}^{2}$$

for m=1,…,N-1. For the acidic tracts and rpL5, the MSD was calculated with respect to all acidic and basic residues, respectively. For all MSDs, we fit the first 30 ps and everything past 200 ps using single exponents to describe the two different regimes. For the MSD with respect to the OD, the region from approximately 80 to 120 ps was found to fit best to a simple diffusion model with $R^{2}=0.9984$. The crossover $t_{D}$ between the super- and sub-diffusive regimes is the median value within the diffusive region of the MSD with respect to the OD. In all cases, the MSD was averaged over five replicates. To calculate the histograms of the exponents of the individual molecules, we modified Eq. (2) to calculate the MSD with respect to each OD rather than averaging over all ODs. We then fit each MSD as before.

### Plotting

To generate the plots, we use Matplotlib^121^ along with Seaborn^122^. Adobe Illustrator^®^ is used to generate the final figures shown.

## Figures and Tables

**Table 1:** Density and temperature for different LJ-phases in LJ-units.

Phase	$\rho^{*}$	$T^{*}$
Vapor	0.01	1.0
Fluid	0.8	1.0
Solid	1.5	0.758
